# Changes in Representation of Thalamic Projection Neurons within Prefrontal-Thalamic-Hippocampal Circuitry in a Rat Model of Third Trimester Binge Drinking

**DOI:** 10.3390/brainsci11030323

**Published:** 2021-03-04

**Authors:** Zachary H. Gursky, Anna Y. Klintsova

**Affiliations:** Department of Psychological & Brain Sciences, University of Delaware, Newark, DE 19716, USA; zgursky@udel.edu

**Keywords:** fetal alcohol spectrum disorders (FASD), thalamus, unbiased stereology, immunohistochemistry, AAV, immunofluorescence, development, connectivity, alcohol

## Abstract

Alcohol exposure (AE) during the third trimester of pregnancy—a period known as the brain growth spurt (BGS)—could result in a diagnosis of a fetal alcohol spectrum disorder (FASD), a hallmark of which is impaired executive functioning (EF). Coordinated activity between prefrontal cortex and hippocampus is necessary for EF and thalamic nucleus reuniens (Re), which is required for prefrontal-hippocampal coordination, is damaged following high-dose AE during the BGS. The current experiment utilized high-dose AE (5.25 g/kg/day) during the BGS (i.e., postnatal days 4–9) of Long-Evans rat pups. AE reduces the number of neurons in Re into adulthood and selectively alters the proportion of Re neurons that simultaneously innervate both medial prefrontal cortex (mPFC) and ventral hippocampus (vHPC). The AE-induced change unique to Re→(mPFC + vHPC) projection neurons (neuron populations that innervate either mPFC or vHPC individually were unchanged) is not mediated by reduction in neuron number. These data are the first to examine mPFC-Re-HPC circuit connectivity in a rodent model of FASD, and suggest that both short-term AE-induced neuron loss and long-term changes in thalamic connectivity may be two distinct (but synergistic) mechanisms by which developmental AE can alter mPFC-Re-vHPC circuitry and impair EF throughout the lifespan.

## 1. Introduction

### 1.1. Gestational Alcohol Exposure Is a Global Public Health Concern, Often Resulting in Impaired Executive Functioning

Alcohol is widely recognized to have negative effects on wellbeing, with any amount of alcohol exposure (AE) ultimately having a net negative impact on health [1]. Considering the critical role that early life experiences have in shaping brain development throughout the lifespan [2,3], fetal AE poses substantial risk to the developing brain [4]. Exposure to alcohol in utero causes a spectrum of physiological, neurological, and behavioral outcomes [5,6], referred to as “Fetal Alcohol Spectrum Disorders” (FASD). One popular model of FASD employs administration of alcohol during a period of rapid brain growth referred to as the “brain growth spurt” (BGS) [7]. The BGS comprises most of the third trimester of human gestation [7], a stage of fetal development when an estimated 8% of pregnancies are impacted by AE [8]. The BGS occurs postnatally in rats (i.e., during the first two postnatal weeks of life), allowing for a preclinical model of late gestational AE with exceptional ability to monitor timing and dose of AE, and to measure blood alcohol concentration achieved from a given administration [9,10]. One of the most prevalent behavioral consequences of developmental AE is altered “executive functioning” (EF) [11,12], as we have previously observed [13], with the most commonly impaired domains being working memory, inhibition, and set shifting [11].

### 1.2. Thalamic Nucleus Reuniens Supports EF by Coordinating Prefrontal Cortex and Hippocampus

EF consisted of multiple domains served by a number of cortical and subcortical brain structures, most notably the prefrontal cortex (PFC) [12]. For instance, the EF domain of spatial working memory relies on coordination of neural activity between hippocampus (HPC) and PFC; encoding of PFC-dependent working memory depends on direct HPC projections to medial PFC (mPFC) [14] and disrupted coherence of HPC-PFC activity (vis-à-vis inactivation of ventral midline thalamus) impairs performance on a working memory-dependent delayed alternation task [15]. The thalamic nucleus reuniens (Re) is responsible for coordinating mPFC and HPC, giving rise to the PFC-Re-HPC circuit involved in many neural processes in charge of EF [16].

Anatomically, the key role of Re in coordinating this network is apparent, as it is recurrently and monosynaptically connected with both mPFC and HPC, and even contains neurons whose axonal projections concurrently innervate both these structures [17,18]. Re also uniquely innervates HPC in a way that allows for precise timing differences between direct (i.e., monosynaptic) Re→mPFC and indirect (i.e., disynaptic) Re→HPC→mPFC input to mPFC. This phenomenon has lately gained attention as a recent study identified that ventral HPC (vHPC) and Re inputs to the prelimbic subregion of mPFC frequently converge and precise timing of these inputs is necessary for plasticity in mPFC [19]. Re is essential for the long-term maintenance of learning-related changes in mPFC anatomy [20], suggesting that disruption of the connectivity and balance of projections within the mPFC-Re-HPC circuit early in life is likely to have deleterious long-term consequences.

### 1.3. Neuroanatomical Alterations to mPFC-Re-HPC Circuitry in FASD

The impact of developmental AE on Re is almost entirely unexplored, with the first of the only two studies to selectively examine this nucleus being published in 2019 [21]. High-dose AE for 6 days during the BGS reduces the number of neurons in Re and reduces the volume of the nucleus while having no effects on these two measures (or others) in the neighboring rhomboid nucleus of the thalamus [21]. This reduction of neurons is likely caused by apoptotic neuron loss triggered by AE, as the same dose of alcohol on a single day during the BGS was sufficient to induce apoptotic cell death 12 h after AE. Moreover, the magnitude of cell death was comparable to the permanent AE-induced neuron loss in Re after either single-day or six-days exposure [21,22]. Despite the robust neuronal loss caused by AE during the BGS in these studies, it is unclear how such neuron loss impacts mPFC-Re-HPC connectivity.

This experiment examines the relationship between AE-induced Re neuron loss and mPFC-Re-HPC neuronal connectivity. We hypothesized that AE during the BGS would alter the pattern of Re projection neurons innervating mPFC, vHPC, or both regions simultaneously. We utilized a widely-used rodent model of third-trimester binge drinking and examined three distinct populations of Re projection neurons using retrograde viral tracing techniques. We include a typically-developing “suckle control” group (SC) in addition to the procedural control “sham intubated” group (SI) to demonstrate that atypical structure and connectivity of Re are caused exclusively by alcohol insult during the BGS, and are not confounded by stresses induced by the method of administration.

## 2. Materials and Methods

**Figure 1:**
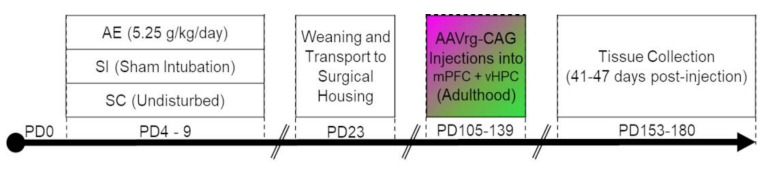
Experimental timeline for the current study.

All procedures performed in this experiment were carried out in accordance with the animal use protocol #1134 approved by University of Delaware (UD) Institutional Animal Care and Use Committee (IACUC), and in accordance with NIH’s Animal Care Guidelines. This study uses a well-characterized rodent model of third trimester-equivalent binge-like AE [21,23,24,25,26,27] to determine the impact of developmental AE on the connectivity of prefrontal-thalamo-hippocampal circuitry (vCA1-Re-mPFC circuit, in particular). A timeline depicting experimental procedures following birth can be found in Figure 1. 

### 2.1. Experimental Subjects

All animals (i.e., Long Evans rats) were bred in-house at UD using first-time mothers placed with experienced male breeders. To address sex as a biological variable, both male and female Long Evans rats were analyzed. All analyses used litter as a random-effect (i.e., “clustering variable”), which accounts for non-independence of observations within litter and allows for generalizability of findings [28], as has been used in past experiments of this nature [21,22] and is discussed more in Section 2.6. This also allowed for the use of a subset of each litter, even if every postnatal treatment was not present (due to loss of individual pups, or uneven sex distribution where such could not be corrected) [29], which is especially critical due to the absence of typically-developing control pups from experimental litters (discussed in the next section). A total of 57 pups from 9 litters were generated and examined (see Table 1).

On PD3, litters were culled to 10 pups (5 males and 5 females, where possible); dams that gave birth to less than 10 pups or did not have 5 male and 5 female pups had pups cross-fostered into their litter to maintain consistent litter size and sex distribution (where possible). A total of 3 pups were cross-fostered: 1 SI female, 1 AE female (littermate to the cross-fostered SI female), and 1 SC male. At this time, all animals had a small amount of non-toxic black India ink subcutaneously injected into the paw pad for identification purposes.

#### 2.1.1. Experimental Postnatal Treatments

This study utilized 3 postnatal treatment paradigms: “suckle control” (SC) typically-developing pups, “sham intubated” (SI) procedural control pups, and alcohol-exposed pups (AE). Postnatal treatments were allocated to pups using a split-litter design where all intubated groups (i.e., SI, AE) were generated within the same litter (“experimental litters”) and the typically-developing SC group was generated from separate (non-experimental, “suckle”) litters, as is common in the literature [23,24]. All mention of “milk” or “milk substitute” refers to a solution identical to that previously described by West, Hamre [30] and widely used in published literature in the decades since.

AE pups were generated from experimental litters. Alcohol delivery occurred in a manner identical to previously described [21,23,24,25,26,27]. Briefly, pups were temporarily removed from the dam (≈10 min) at 9:00AM for daily weighing and first alcohol administration. The necessary dose of 11.90% vol/vol ethanol in milk substitute was calculated for each individual pup daily such that the pup received 5.25 g/kg/day of EtOH over the first two administrations (i.e., 2.625 g/kg/dose at 9 AM and 11 AM), as is common in this paradigm [26,31,32]. At 9:00 AM, 11:00 AM, and 1:00 PM (additionally, 3:00 PM on PD4 only), exactly 2 h apart ± 5 min, each litter of pups was removed from their dam for intubation and/or administration of milk substitute. Intubation for each pup consisted of coating the tip of a plastic feeding tube (Instech, Laboratories, product # FTP-22-38) with corn oil and gently moving the tube down the esophagus before administering the appropriate solution. This process took approximately 10–15 seconds per pup. All AE animals received supplemental milk doses at systematic intervals (2 h after each final daily dose of AE) throughout the period of alcohol administration to minimize potential reductions in body weight and minimize confounding gross anatomical underdevelopment, as is frequently reported in past studies [33,34]. This is essential as intoxicated pups show temporary inability to suckle from their respective dam and need this to supplement their growth in the absence of suckling during intoxication. Analysis of weight during treatment and in adulthood can be found in Supplementary Material (Figure S1, Table S1), and is discussed in the context of relevant literature (e.g., [32,35,36]) in Section S.1.

In addition to intubation, AE pups underwent one blood collection from the tail vein to assess peak blood alcohol concentration (BAC). Exactly 90 min after each animal’s final alcohol administration for the day on PD4, pups were briefly removed from the dam, and had 60 µL of blood from a tail clip collected into a heparinized capillary tubes. Blood was immediately transferred to a sterile centrifuge tube and stored on ice. The subsequent steps of blood sample preparation and BAC analysis are described in further detail in Section 2.1.2.

SI pups were also generated from experimental litters. As an appropriate procedural control, SI pups underwent all procedures that were performed on AE pups: SI pups were briefly removed from their dam, weighed daily at 9AM, intubated alongside AE pups at the same intervals, and underwent blood collection (bloods were not analyzed). All SI intubations occurred without administration of any liquid (milk, EtOH). The lack of liquid administration to SI pups is critical to minimize the potentially confounding variable of weight difference between AE and SI groups during development [24].

SC pups were generated from suckle litters. Typically-developing SC control pups were briefly removed from their dam (≈5 min) to be weighed daily at 9AM and returned to the dam. SC pups remained undisturbed from PD4-9 except for this daily weighing.

#### 2.1.2. Blood Alcohol Concentration Analysis

Blood samples for BAC analysis were collected at exactly 90 minutes following the final alcohol dose on PD4, the time at which BAC is highest [9]. Peak BAC is a robust predictor of alcohol-induced damage to the central nervous system, more so than total amount of alcohol administered [37], so this measure is reported within each experiment to validate level of alcohol exposure.

Immediately after blood collection from pups, samples were centrifuged at 15,000× *g* at 4 °C for 25 min. Supernatant plasma was collected and stored at −20 °C until BAC analysis using an Analox GL5 Analyzer (Analox Instruments, Boston, MA, USA) and the manufacturer’s protocol for alcohol content analysis. Mean (±SEM) peak BAC achieved on PD4 in the current experiment was 205 ± 39 mg/dL.

#### 2.1.3. Weaning

Following experimental manipulation on PD4-9, all animals were left undisturbed until PD23. On PD23, animals were weaned into cages of 2–3 same-sex non-littermates of mixed postnatal treatments, as has been done in past studies using this paradigm [38]. These cages were immediately transported to housing for surgical procedures, to allow for necessary acclimation.

### 2.2. Stereotaxic Surgery

Aseptic stereotaxic surgical protocol was approved by veterinarian review followed by UD IACUC and UD biosafety committee approval. All stereotaxic injections occurred in adulthood, between PD105-139 (Figure S2, Table S2). Animals were left undisturbed except for daily veterinary monitoring for 41–47 days to allow sufficient fluorophore expression from AAVrg-CAG infection. Variability of incubation time was not associated with any postnatal treatment or sex (see Supplementary Material: Section S.2.1). No veterinary complications were observed in any of the 57 animals.

To begin, animals were anesthetized and maintained under anesthesia using inhalable isoflurane ranging from 4% to 0.5%, had body temperature maintained during anesthetization using a heating pad, and eye lubrication applied.

Following sterilization and incision at the surgical site, burr holes were drilled into the skull at 3.00 mm anterior, −1.50 mm lateral (i.e., left) and 5.70 mm posterior, −5.40 mm lateral (i.e., left) relative to bregma to allow access to the mPFC and vHPC, respectively, and were immediately covered until injection. These coordinates are based on, but modified slightly from, Varela, Kumar [17], due to a handful of pilot targeting injections (data not shown). Most studies that record from HPC to examine coordination of HPC-mPFC activity in vivo examine the dorsal portion of CA1 (e.g., [15,39]), and some record from both dorsal and ventral CA1 (e.g., [14,40]). This experiment injected AAVrg-CAG into ventral CA1 due to the fact that Re densely innervates vCA1 (contrasting to the neighboring rhomboid nucleus, which minimally does) and that vCA1 neurons that monosynaptically project to mPFC are responsible for HPC-mPFC coordination [40,41]. The mPFC injection site was approached at a 16° angle to minimize risk of damage to the superior sagittal sinus along the midline. Due to mechanical restrictions of the stereotax given the 16° angle of PFC injections, all injections were located in the left hemisphere; mPFC injection always occurred first (followed by vHPC injection).

400 nL of a single AAVrg-CAG per site was withdrawn into a 1 µL Hamilton “Neuros” syringes (Hamilton Company, Product # 65458-01 with custom 45° tip angle) attached to a programmable syringe pump (Harvard Apparatus, product # 70-4507). The syringe was slowly lowered into the brain at a rate of approximately 1.0 mm/min. Before and after injection, the syringe remained in place at the injection site for 5 min to reduce diffusion backward along the syringe tract and allow for complete diffusion of AAVrg-CAG into the brain from the syringe tip [42]. 400 nL of AAVrg-CAG was injected at a rate of 100 nL/min over 4 min using the programmable syringe pump. The injections were successful in targeting prelimbic PFC in mPFC and CA1 in vHPC (the observed location according to Paxinos & Watson [43] and diffusion of AAVrg-CAG at each site is examined in more detail and compared to relevant literature [44,45] in the Supplementary Material: Section S.2.2., Figure S2, Tables S3–S5). The syringe was then removed from the brain, and this process was repeated with a different, but identical, syringe for the second injection to eliminate any possibility of cross-contamination between different AAVrg-CAG injections. Fluorophore-injection site pairings were randomly counterbalanced between postnatal treatment and sex.

#### AAVrg-CAG Characteristics

The AAV constructs used in this experiment were acquired from the non-profit plasmid repository, Addgene (Watertown, MA, USA), and expressed either tdTomato (Product # 59462-AAVrg, produced from pAAV-CAG-tdTomato (codon diversified)) or GFP (Product # 37825-AAVrg, produced from pAAV-CAG-GFP). Both of these plasmid constructs were deposited in the Addgene repository by Edward Boyden. Both constructs were utilized in identical titer, with viral particles concentrated at 5.0 × 10^12^ viral genomes (vg)/mL. These AAVrg-CAG constructs have been demonstrated to produce robust retrograde labeling of projection neurons in the rat brain after incubation for at least 3–5 weeks [46].

### 2.3. Tissue Fixation

Brain tissue was fixed and extracted in adulthood, approximately 6 weeks after AAVrg-CAG injection (PD153-180). The amount of time between AAVrg-CAG injection and tissue fixation did not differ between postnatal treatments or sexes (see Supplementary Material: Section S.2.1). For tissue collection, each animal was weighed, then deeply anesthetized using a veterinarian-approved dose of ketamine and xylazine delivered via intraperitoneal injection. Brains were perfused with approximately 100 mL of 0.1 M phosphate buffered saline (PBS; pH = 7.20), followed by 100 mL of 4% paraformaldehyde (PFA) in 0.1 M PBS. Brains were postfixed in 4% PFA for 48 h, then equilibrated in 30% sucrose in 4% PFA three times.

Cerebellum and brain stem were removed from the midbrain and forebrain. The remaining portion of each brain was then exhaustively sectioned in a coronal plane at 40 µm in a cryostat (CM3050S; Leica Biosystems, Wetzlar, Germany) at −20 °C. Serial sections were collected in a sucrose and ethylene glycol-based cryoprotectant solution and stored at −20 °C until immunofluorescence (IF) processing.

### 2.4. Immunofluorescent Labeling

To examine the total number of projection neurons in Re, we visualized neuronal nuclei using primary antibody against NeuN (Neuronal Nuclei, or Hexaribonucleotide Binding Protein-3) [47] and expression of fluorescent labels from AAVrg-CAG constructs. Hoechst33342 was also applied to all tissue but was only visualized to histologically locate and quantify injection location and diffusion in mPFC and vHPC (i.e., not in examination of Re).

**Figure 2:**
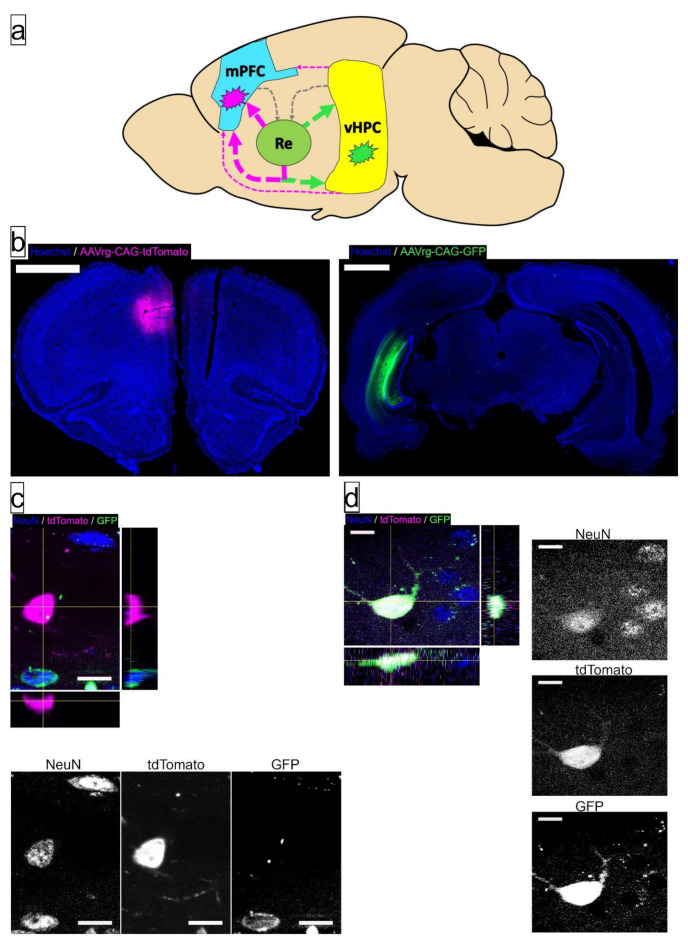
Schematic representation and visualization of retrograde-labeling AAVrg-CAG injections into medial prefrontal cortex (mPFC), ventral hippocampus (HPC), and exemplar labeled cells in thalamic nucleus reuniens (Re). (**a**) A schematic indicating the approximate sites of AAV injection (indicated by magenta and green explosion symbol). Connectivity within mPFC-Re-HPC circuitry is presented as arrows, with the large, colored, arrows leaving Re as the population of projection neurons examined. Pairing of injection site and fluorophore was counterbalanced between postnatal treatment and sex. Connectivity diagram is derived from [48,49]; (**b**) Representative sections containing the center of the injection site into mPFC (left) and HPC (right). Scale bars = 2 mm; (**c**) Examples of Re projection neurons (NeuN^+^) projecting to the injection site of AAVrg-CAG-tdTomato (NeuN^+^/GFP^−^/tdTomato^+^, in crosshairs), AAV-CAG-GFP (NeuN^+^/GFP+/tdTomato+, bottom left), or neither injection site (NeuN^+^/GFP^−^/tdTomato^−^, top right). Bars on right and bottom of image show Y-Z and X-Z planes of image stack, respectively, while the panels below show each color channel in isolation. Scale bar = 15 µm; (**d**) A subpopulation of neurons in Re project to both mPFC and HPC, as indicated by expression of fluorophores from both injection sites (NeuN^+^/GFP^+^/tdTomato^+^, in crosshairs). Bars on right and bottom of image show Y-Z and X-Z planes of image stack, respectively, while the panels on the right show each color channel in isolation. Scale bars = 15 µm.

First, starting at a random starting point in the frontal lobe, every 6th serial coronal section was systematically selected for IF labeling and analysis in this experiment. In the coronal plane, there was no overlap between the mPFC injection site, Re, and vHPC injection site. As a result, mPFC and vHPC sections were immediately counterstained with Hoechst33342 and slide mounted for analysis (representative sections are presented in Figure 2). Sections containing Re underwent IF labeling for NeuN, identical to that used in [21].

Briefly, tissue was first washed in deionized H_2_O (dH_2_O), followed by 0.1M tris buffer solution (TBS; pH = 7.40). Nonspecific antibody binding was inhibited using a NDS-TX-TBS blocking solution consisting of 3.0% normal donkey serum (NDS; Millipore Sigma, S30) and 0.5% Triton X-100 (ThermoFisher Scientific, Product # 85111) in 0.1 M TBS for 2 h. After blocking solution, tissue was immediately transferred to the primary antibody, Mouse anti-NeuN (Millipore Sigma, MAB377, 1:500 dilution) in NDS-TX-TBS blocking solution. Negative-control sections were incubated in NDS-TX-TBS blocking solution without primary antibody. Tissue was incubated at 4 °C for approximately 40 h. After primary antibody incubation, tissue was washed in 0.1 M TBS before being transferred into secondary antibody (Donkey anti-mouse conjugated with Alexa Fluor647, Jackson ImmunoResearch, 715-605-151, 1:250 dilution) in NDS-TX-TBS blocking solution for 3 h at room temperature. After secondary antibody incubation, tissue was washed in 0.1 M TBS followed by 0.1 M PBS. Lastly, sections were incubated in 0.4 µg/mL Hoechst33342 in PBS before final washes in 0.1 M PBS and transfer to microscope slides, where a coverslip was applied with gelvatol mounting medium [50]. Slide-mounted tissue dried at room temperature for approximately 24 h, then was stored at 4 °C until imaging, at least 7 days after mounting medium and coverslips were applied to allow appropriate hardening of mounting medium. Representative image stacks acquired for analysis following IF labeling (i.e., visualizing GFP, tdTomato, and NeuN-bound Alexa Fluor647) can be observed in Figure 2.

### 2.5. Fluorescence Microscopy

All microscopy utilized a Zeiss AxioImager M2 with Apotome and Colibri 7 LED illumination (Carl Zeiss AG, Oberkochen, Germany). Use of a Zeiss apotome for structured illumination fluorescent imaging and a high-numerical aperture (NA) 63× oil immersion objective (NA = 1.40, Plan Apochromat, product # 420780-9900-000) allow for acquisition of image stacks where each individual image has high axial resolution, resulting in accurate estimation of volume and neuron number in Re. Fluorophores in tissue underwent selective and sensitive excitation by combining adjustable-intensity light emitting diodes with single bandpass filter cubes for DAPI/Hoechst (Carl Zeiss AG, Oberkochen, Germany, Filter set 96 HE, Product # 489096-9100-000), GFP/Alexa Fluor 488 (Carl Zeiss AG, Oberkochen, Germany, Filter set 38 HE, Product # 489038-9901-000), tdTomato/Alexa Fluor 568 (Carl Zeiss AG, Oberkochen, Germany, Filter set 43 HE, Product # 489043-9901-000), and Alexa Fluor 647 (Carl Zeiss AG, Oberkochen, Germany, Filter set 50, Product # 488050-9901-000, Zeiss), and imaged by a high-sensitivity monochrome camera (ORCA-Flash4.0 LT+ Digital CMOS camera, Hamamatsu Corporation, Middlesex, NJ, USA).

Volume estimation using Cavalieri estimation occurred using the same system mentioned in the previous paragraph, equipped with a 5x non-immersion objective (NA = 0.16, Plan-Neofluar, product # 420330-9901-000) without the use of the Apotome.

### 2.6. Unbiased Stereological Estimation

A popular and rigorous method of quantification for parameters such as number and volume is unbiased stereological estimation [51,52]. Unbiased stereological estimation is a process by which properties of a brain structure can be determined through exhaustive systematic random sampling of that structure. Using Stereo Investigator 2019 (MBF Bioscience, Williston, VT, USA) software, we were able to examine various properties of the mPFC-Re-vHPC circuit and quantify the reliability of these data using a coefficient of error (CE) [53], as presented in Table 2.

Adherence to the principles of unbiased stereology results in estimates of total number (e.g., total volume of a structure, total number of cells) rather than relative measurements (e.g., cell density, or volume per section). Examining total number rather than density measurements is critical in understanding potentially nuanced alterations in the brain, especially those caused by an underlying neuropathology or neurodevelopmental insult with corresponding or potential change in the volume of brain structure [51]. We utilized two common stereological probes: the optical fractionator probe, which estimated the total number of various cell types within Re, and the Cavalieri estimator (point-counting) probe, which estimated total volume of spread of an injected substance (i.e., AAVrg-CAG injections). 

Due to the unilateral nature of injections, and well characterized ipsilateral nature of the mPFC-Re-HPC circuit of interest [15,17,55], all stereological examination of Re only utilized the left half of Re, as the structure is easy to divide in half by visual inspection due to its proximity to the midline 3rd ventricle. The observed mean Gundersen coefficients of error [53,54] for all stereological probes can be found in Table 2, and are appropriately below the widely-acceptable threshold for robust and reliable estimates (i.e., a CE of 0.100).

The volume of diffusion of AAVrg-CAG injection was estimated using the Cavalieri estimation probe, with every 6th serial coronal section of mPFC and vHPC prepared and examined. Systematic random sampling of the area of each injection within each section involved analysis of a systematic grid of points in 100 µm × 100 µm intervals.

Total neuron number in left Re was estimated using the optical fractionator probe, with every 6th serial coronal section of Re prepared and examined. Systematic random sampling of the area of each section involved analysis of a 40 µm square counting frame spaced along an 80 µm square systematic grid. This resulted in an analysis of 25% of the area of Re in each section. The thickness of the optical disector (probe from which the counting frame is derived) was 10 µm, with guard zones of 4 µm to allow for unevenness of the top of each section that may arise during sectioning, tissue preparation, and slide-mounting. Thickness of each section was measured at every 10th counting site (resulting in approximately 15–30 thickness measurements per brain).

### 2.7. Statistical Analyses

An α = 0.050 level of significance was used for all statistical analyses. All statistical analyses were performed using the R programming language version 4.0.2 [56] in the RStudio integrated development environment [57]. Scripts loaded the following packages: lme4 [58], lmerTest [59], mediation [60], readxl [61], sjPlot [62], and tidyverse [63].

To account for potential source of variation between-litter [28], all analyses were performed as linear mixed-effects models (LMMs) using the “lmer” function in “lmerTest” package with litter included as a random effect. For all analyses, the procedural control group (i.e., the SI treatment group) was considered the baseline reference group for postnatal treatment as a fixed effect and females were considered the baseline reference group for biological sex as a fixed effect. The decision to make the procedural control group the reference group for analysis comes from the recognition that the causal impact of alcohol can only be considered as a difference from the procedural control group, as there are procedural confounds preventing the interpretation of differences between AE groups and typically developing groups (nevertheless, we observed no differences between the procedural control group and the typically developing groups in any neuroanatomical measures and only subtle differences in weight gain during the early postnatal period). All analyses, including simulation-based analyses (i.e., mediation analysis) were scripted and utilized a randomly generated numerical seed, to allow for reproducible analysis.

Comprehensive presentation of exact statistical values can be found in the results section in the form of regression tables (including a listing of all predictors, estimates of effects, 95% confidence intervals, *p*-values, degrees of freedom, and metrics of random effects). As a result, the text of the results section only addresses key findings for sake of brevity. Sex as a biological variable was not associated with any measured neuroanatomical outcomes. As a result, visualizations of data are combined between sex within each postnatal treatment group and discussion of sex is not included in this Results section Sample sizes separated by experiment, sex, and postnatal treatment, are presented in Table 1.

## 3. Results

### 3.1. High-Dose AE Reduces Re Neuron Number and Volume

**Figure 3:**
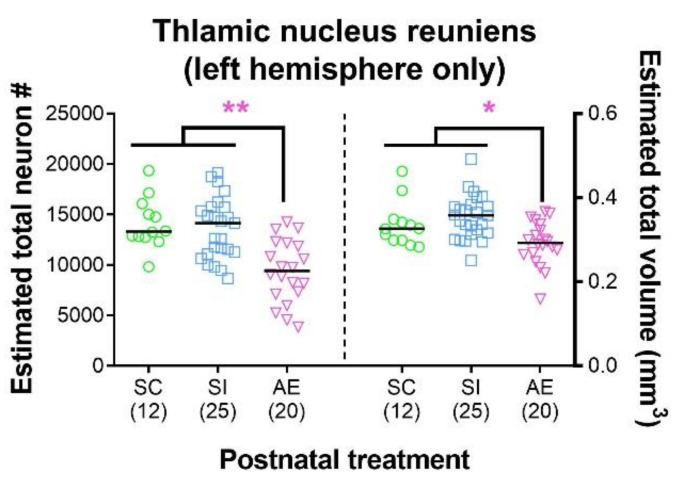
High-dose alcohol exposure (AE) during the brain growth spurt (BGS) results in reduced number (#) of neurons in thalamic nucleus reuniens (Re) and a reduction in the total volume of Re. The AE postnatal treatment group had significantly lower number of neurons in Re (left) and significantly reduced total volume of Re (right), consistent with past studies [21,22]. Individual data points represent one animal and are superimposed over bars representing mean within that postnatal treatment. Data include both male and female animals, which are not differentiated due to the lack of sex effects. Sample sizes are given in parentheses below each treatment group. * *p* ≤ 0.050, ** *p* ≤ 0.010. For exact statistical values, see Table 3.

We examined only the hemisphere of Re ipsilateral to the mPFC and vHPC injection sites (i.e., the left hemisphere), as the mPFC-Re-vHPC circuitry of interest is ipsilateral [15,17,55]. These data are presented in Figure 3, and greater statistical detail can be found in Table 3. AE caused significant reductions in both neuron number (*p* = 0.001) and volume (*p* = 0.019) in both sexes relative to both control groups, a pattern of Re damage consistent with past reports [21,22]. We observed no significant differences between the SC typically developing control group and the SI procedural control group either neuron number (*p* = 0.790) or volume of Re (*p* = 0.217).

### 3.2. AE Selectively Increased the Proportion of Re Projection Neurons That Concurrently Innervate mPFC and vHPC, but Not Those That Innervate either Region Individually

**Figure 4:**
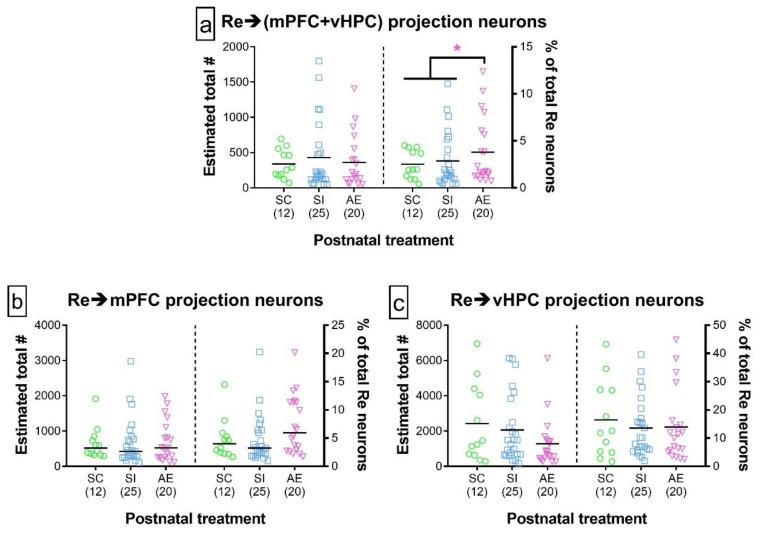
Quantification of Re projection neurons that simultaneously innervate mPFC and/or vHPC. (**a**–**c**) Total number of Re projection neurons (left of each panel) and the proportion of total Re neurons comprised of this class of projection neuron (right of each panel) that innervated mPFC and vHPC concurrently (**a**), mPFC alone (**b**), or vHPC alone (**c**). While the total number of each class of projection neuron was not influenced by postnatal treatment, the proportion of Re→(mPFC + vHPC) neurons (relative to total neurons in Re) was significantly increased in the AE treatment group. For all panels in this figure, individual data points represent one animal and are superimposed over bars representing mean within that postnatal treatment. Data include both male and female animals, which are not differentiated due to the lack of sex effects. Sample sizes are given in parentheses below each treatment group. * *p* ≤ 0.050. For exact statistical values, see Table 4, Table 5 and Table 6 (and Tables S6–S8 for injection parameter-controlled values).

The proportion of Re neurons innervating both mPFC and vHPC (“Re→(mPFC + vHPC)”) was significantly increased in the AE group relative to both control groups (*p* = 0.021), while the proportion between control groups was not different from each other (*p* = 0.963). No influences of AE were observed in proportion of Re neurons that only innervate mPFC (*p* = 0.396) or vHPC (*p* = 0.981). There were no AE-induced differences in the total number of Re projection neurons of any of these three subtypes (*p*’s > 0.1243). These findings hold true even when correcting for the diffusion of AAV injection at each injection site, the number of incubation days of AAVrg-CAG, and the pairing between fluorophore and injection site (see Supplementary Material: Section S.2.3, Tables S6–S8). These data are presented in Figure 4. Representative injections of AAVrg-CAG are presented in Figure 2, accompanied by examples of retrograde labeling.

### 3.3. AE-Induced Changes in Re→(mPFC + vHPC) Projection Neuron Representation Are Not Mediated by AE-Induced Re Neuron loss

To determine whether the increase in the proportion of Re→(mPFC + vHPC) projection neurons was the result of a decrease in total Re neuron number, or an AE-induced change in their representation independent of Re neuron number, we performed mediation analysis. This method allowed us to statistically determine whether an outcome (i.e., proportion of Re→(mPFC + vHPC) projection neurons, in this experiment) was better predicted directly by the presence of a treatment (i.e., AE), by the treatment’s impact on a third (“mediating”) variable (i.e., total neuron number in Re), or by both the direct and mediated effects. We used 100,000 bootstrapped simulations, as has been appropriate in past studies [22,64].

**Figure 5:**
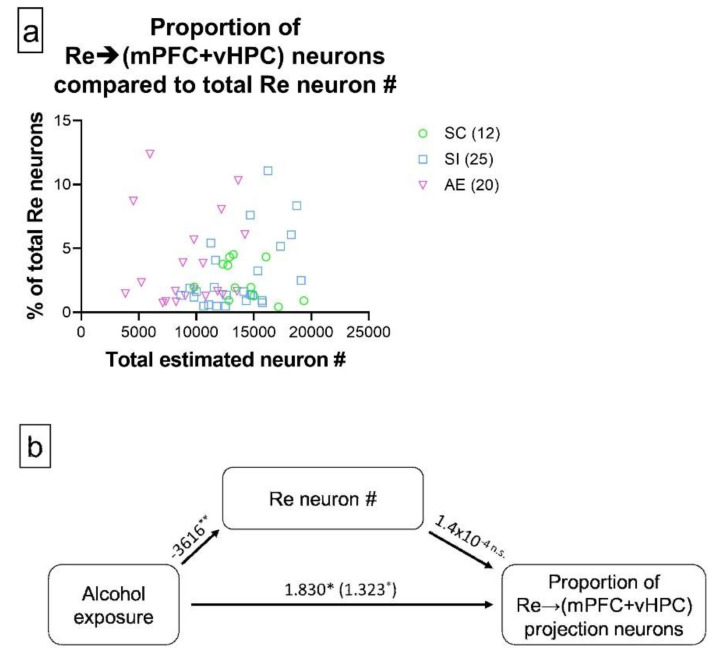
Neuron loss did not mediate the AE-induced increase in proportion of Re neurons that concurrently innervate mPFC and vHPC. (**a**) Scatterplot showing the relationship between total neuron number in Re and proportion of Re→(mPFC + vHPC) projection neurons; (**b**) Schematic representation of the mediation analysis between AE, Re neuron number, and proportion of Re→(mPFC + vHPC) projection neurons. The total effect of AE (in % of neurons) is shown in parentheses, while the direct effect is listed immediately next to the total effect (also in % of neurons). While the relationship between AE and Re neuron number was significant (estimate given in number of neurons), there was no significant association between Re neuron number and proportion of Re→(mPFC + vHPC) projection neurons, resulting in a lack of mediating effect. Data include both male and female animals, which are not differentiated due to the lack of sex effects. Sample sizes are given in parentheses next to each treatment group in the panel (**a**) legend. * *p* ≤ 0.050, ** *p* ≤ 0.010, n.s. = not statistically significant. For exact statistical values, see Section 3.3.

Our analyses indicated that the significant total effect of AE on the proportion of Re→(mPFC + vHPC) projection neurons (estimate = 1.323, 95% CI = 0.009 to 2.640, *p* = 0.048) was not mediated by AE-induced reductions in Re neuron number (estimate = −0.507, 95% CI = −1.574 to 0.450, *p* = 0.301). Instead, the total effect could be entirely accounted for by the direct effect that AE has on the outcome (estimate = 1.830, 95% CI = 0.242 to 3.430, *p* = 0.023), indicating that changes in the representation of Re→(mPFC + vHPC) projection neurons in Re occur independently of AE-induced Re neuron loss. The diagram of all individual data points can be found in Figure 5a, and the appropriate schematic demonstrating the mediation analysis can be observed in Figure 5b.

## 4. Discussion

### 4.1. Summary of Key Findings

The current study examined whether neuron loss in Re caused by AE during the BGS alters neuronal connectivity of mPFC-Re-vHPC circuitry throughout life. High-dose AE during the BGS selectively increased the proportion of Re neurons that innervate both mPFC and vHPC concurrently (but not either region individually) in adulthood, despite a lack of change in total number of Re projection neurons within the mPFC-Re-vHPC circuit in adulthood. This increase in representation of this class of projection neuron was not mediated by changes in neuron number, suggesting that this AE-induced change in mPFC-Re-vHPC circuit occurs independent of damage to Re via apoptotic neuron loss.

There were no statistically-significant differences between SC (i.e., typically-developing control group) and SI (i.e., procedural control group) groups on any measures, indicating that the observed changes in the AE group were not caused by developmental stress, and are consequences of developmental AE alone.

### 4.2. The Pressing Need to Identify Primary versus Secondary Consequences of Developmental AE

While it is commonly recognized that developmental AE damages the HPC, and more recently recognized that PFC is also damaged, it had been unclear what damage is primary (i.e., directly caused by) AE, and what changes are secondary (i.e., not directly caused by AE, but is the consequence of AE-induced brain changes) [65]. Although the brain is vulnerable to processes of alcohol-induced apoptotic cell death through various developmental processes including synaptogenesis [66], such damage is considered a primary consequence of developmental AE. All regions in the mPFC-Re-HPC circuit are vulnerable to this (direct) form of AE-induced damage (i.e., apoptotic cell death, which occurs within just hours of AE) during the first two postnatal weeks [22,66].

In contrast, it remains an important topic of discussion to understand when the changes in the proportion of Re neurons that collaterally innervate HPC and PFC (and likely play a key role in coordinating circuit activity, per Varela, Kumar [17]) arise following developmental AE. If the change in connectivity occurs early in life, it is possible that behaviorally relevant changes in HPC and PFC could result from imbalance of activity from different inputs and the absence of orchestrated network activity throughout the lifespan (this would be a strong secondary effect of developmental AE on PFC structure and function). It is important to note that increased representation, hyperconnectivity, or increased synchrony is not necessarily beneficial, as both upregulation and downregulation of activity in thalamic projection neurons targeting mPFC and/or HPC result in atypical function of mPFC-Re-HPC circuitry and related behaviors [67]. 

It is also unclear, at this point, why the representation of only a single class of Re projection neurons was changed by developmental AE despite the overall loss of Re neurons in the current study. While the current study does not examine the functional consequences of changes in the balance of Re excitatory projections to different structures, it identifies the need for in vivo electrophysiological recording to identify potential functional correlates of shifting representation of Re microcircuits in future studies.

This hypothesis that a behavioral circuit relies on appropriate balance between multiple functional subunits, and that minor disruptions to coordinated activity earlier in life may drive long-term changes in the function of individual structures was proposed by McDonald, Devan [68] as a corollary to “interacting memory systems theory” (IMST). Longitudinal analysis of coordinated activity between multiple brain regions through electrophysiological recording or functional neuroimaging (which is a noninvasive but an indirect measure of neural activity, which has been performed in humans to identify relationships between structure and function of EF networks during development [69]) would help clarify the ontogeny of disrupted balance between Re microcircuits.

### 4.3. A Proposed Cellular Mechanism by Which Re Damage May Impair PFC Function through Altered Timing of HPC and Re Input

A critical secondary influence of Re on PFC and HPC function throughout life is the ability of the brain to coordinate HPC-PFC activity. Changes in timing between mono- (Re→mPFC) and disynaptic (Re→vCA1→mPFC) inputs to mPFC could result in impairments in input timing-dependent plasticity (ITDP) (e.g., [70,71]) and ultimately learning. In a recent first investigation of this phenomenon, Banks, Warburton [19] demonstrated that the majority of layer 5 pyramidal neurons in the prelimbic subregion of mPFC receive input from both Re and vCA1. The same study further demonstrated (in ex vivo brain slices) that precise temporal coordination of vCA1→mPFC and Re→mPFC inputs resulted in NMDA-mediated ITDP. Paring and induction of ITDP occurred following stimulation in the theta frequency range, (a correlate of working memory when Re coordinates HPC and mPFC at theta [15]).

Given this precise regulation of differential timing between HPC and Re inputs to mPFC necessary for ITDP (approximately 10ms according to Banks, Warburton [19]), it follows that damaging Re and altering the proportion of projection neurons in Re that collaterally innervate vCA1 and mPFC would be likely to influence EF through impaired mPFC function (e.g., [13,20]). This would also account for sparing of orbitofrontal function of any behaviorally-relevant wave of secondary damage from developmental AE (i.e., lack of reversal learning deficit in [13]), as there have been no observations of Re neurons collaterally projecting to both vHPC and orbitofrontal cortex.

### 4.4. Limitations of the Current Study

There is evidence (albeit limited) that the mPFC-Re-HPC system is at a similar stage of development and susceptibility to AE (which targets synaptogenic neurons [66]) as PFC spine density and synaptogenesis are similar between this model and the human at the aforementioned developmental stages [72,73]. While there is currently no literature comparing development of Re (selectively within thalamus) between humans and rodents, limiting the translatability of the current study, the critical role of Re in rodent EF [15,16,19,20,67,74,75] and its susceptibility to developmental AE [21,22] results in a need to address this gap in knowledge.

Our procedural control group demonstrated that there was no impact of stress resulting in changes in Re relative to typically-developing animals (i.e., the “SC” group). While this was the case, it is possible that there was an interaction between early life stress and developmental alcohol exposure. While this interaction is of significant interest to the field [76], it has only been studied intensely and mechanistically by separating the two insults (i.e., prenatal AE and postnatal early life stress) [77]. The interaction between developmental stressors and pharmacological insult is of pressing clinical importance, but is beyond the scope of the current study.

While the current study constitutes the first one to examine mPFC-Re-HPC circuitry in a rodent model of FASD, the methodological limitations of the utilized retrogradely-labeling AAV must be addressed. The AAVrg-CAG used is taken up at the axon terminal and results in expression of a fluorescent marker in the soma of the neuron, making it unclear exactly what cells in the injection site the projection neurons synapse on, which also limits knowledge regarding other qualities of the synapse that may be important (e.g., maturational state or morphology of dendritic spines, expression of different classes of neurotransmitter receptors, etc). Future examination using complementary anterograde and retrograde labeling in the circuit of interest will result in a higher fidelity dissection of microcircuits within the mPFC-Re-HPC circuit, and can be combined with functional recording techniques (e.g., in vivo electrophysiology) to provide comprehensive structure-function characterization.

The exact cause of the increase in Re→(mPFC + vHPC) projection neuron representation is unclear, due to the lack of significant change in either projection neuron population and independence from total Re neuron number. As our sample size would have been able to detect a large change resulting from remapping of only one population, it is likely that this is caused by subtle (i.e., low magnitude/small effect size) changes in connectivity distributed across multiple neuronal populations. Replication of our observed relationship between Re→(mPFC + vHPC) neuron number and connectivity of Re with mPFC and vHPC alone is critical in determining the source of this remapping and effect of AE on circuit-wide dynamics of the mPFC-Re-vHPC network.

## 5. Conclusions

Developmental AE consistently and substantially damages Re, causing a reduction in the number of neurons and the size of the nucleus. The selective impact of AE on neurons that simultaneously innervate mPFC and vHPC (rather than neurons that only innervate one of those regions) gives rise to the hypothesis that impaired coordination of PFC with HPC is likely to impair EF in cases of FASD, rather than outright reduction in connectivity with either (or both) structure(s) outright.

The current study provides a small but essential foundational snapshot into the ways in which thalamic damage may contribute to atypical brain development following developmental AE. While Re clearly plays a critical role in EF, it is even more important to look at the whole of the mPFC-Re-HPC circuit (structurally and functionally) when assessing behavioral outcomes, as the appropriate integration of these structures seems to be quite delicate in neurodevelopmental disorders, akin to the hypotheses and corollaries established with IMST. The multiple ways in which Re is consistently altered by developmental AE demonstrate an imperative for identifying the differences between primary and secondary sources of hippocampal and prefrontal dysfunction in FASD.

## Figures and Tables

**Table 1:** Sample sizes for each postnatal treatment group in this study. SC: “suckle control” (typically-developing control group), SI: “sham-intubated” (procedural control group), AE: “alcohol-exposed” (5.25 g/kg/day) experimental treatment group.

Postnatal Treatment	Sex	Number of Litters Represented	Total Sample Size
SC	Female	2	8
SC	Male	2	4
SI	Female	7	14
SI	Male	7	11
AE	Female	5	12
AE	Male	6	8

**Table 2:** Sample sizes and mean Gundersen (m = 1) coefficients of error (CEs) [54]. Stereological estimates included diffusion of AAVrg-CAG injections in mPFC and vHPC, and Re neuron number in the hemisphere ipsilateral to AAV injections. Estimates are commonly accepted as robust and reliable if CE values are below 0.100 [53].

Postnatal Treatment	Sex	Sample Size	mPFC Injection Diffusion Volume (Mean CE ± SEM)	vHPC Injection Diffusion Volume (Mean CE ± SEM)	Re Neuron # (Mean CE ± SEM)
SC	Female	08	0.075 ± 0.010	0.048 ± 0.004	0.074 ± 0.002
SC	Male	04	0.068 ± 0.021	0.044 ± 0.006	0.070 ± 0.000
SI	Female	14	0.074 ± 0.010	0.057 ± 0.010	0.074 ± 0.002
SI	Male	11	0.063 ± 0.007	0.052 ± 0.008	0.071 ± 0.003
AE	Female	12	0.071 ± 0.013	0.052 ± 0.007	0.084 ± 0.005
AE	Male	08	0.056 ± 0.007	0.060 ± 0.019	0.091 ± 0.005

**Table 3:** Regression table for Re neuron number and Re volume, as visualized in Figure 2 and quantified in Figure 3. Table was generated using the “tab_model” function (df method = Satterthwaite) from the “sjPlot” package on “lmer” objects from the “lmerTest” package. Significant predictors (i.e., *p* ≤ 0.050) are indicated by bold *p*-values.

	Re Neuron Number				Re Volume (mm^3^)			
Predictors	Estimates	95% CI	*p*	*df*	Estimates	95% CI	*p*	*df*
(Intercept)	13884.911	12109.685–15660.138	**<0.001**	17.265	0.352	0.324–0.379	**<0.001**	51.000
SC	−461.776	−3780.639–2857.086	0.790	10.724	−0.029	−0.074–0.016	0.217	51.000
AE	−3615.875	−5673.838–−1557.913	**0.001**	45.344	−0.050	−0.090–−0.010	**0.019**	51.000
Male	−125.126	−2216.416–1966.163	0.907	44.351	0.005	−0.036–0.046	0.815	51.000
SC × Male	2800.211	−1040.931–6641.354	0.160	45.444	0.037	−0.038–0.112	0.340	51.000
AE × Male	−1074.289	−4294.209–2145.631	0.516	46.255	−0.031	−0.093–0.032	0.339	51.000
**Random Effects**								
σ^2^	6925310.96				0.00			
τ_00_	2151974.33 _Litter_				0.00 _Litter_			
ICC	0.24							
N	9 _Litter_				9 _Litter_			
Observations	57				57			
Marginal *R*^2^/Conditional *R*^2^	0.336/0.493				0.255/NA			

**Table 4:** Regression table for Re→(mPFC + vHPC) projection neurons, as visualized in Figure 2 and quantified in Figure 4. Table was generated using the “tab_model” function (df method = Satterthwaite) from the “sjPlot” package on “lmer” objects from the “lmerTest” package. Similar table controlling for injection properties (diffusion of AAVrg-CAG at the injection sites (fixed effects), the amount of incubation time following AAV injection (fixed effect), and the fluorophore-site pairing (random effect)) can be found in the Supplementary Material (Section S.2.3). Significant predictors (i.e., *p* ≤ 0.050) are indicated by bold *p*-values.

	Number of Projection Neurons: Re→(mPFC + vHPC)				% of total Re Neurons: Re→(mPFC + vHPC)			
Predictors	Estimates	95% CI	*p*	*df*	Estimates	95% CI	*p*	*df*
(Intercept)	397.355	117.713–676.997	**0.005**	49.000	2.864	0.725–5.002	**0.009**	49.000
SC	9.298	−544.136–562.733	0.974	49.000	0.102	−4.196–4.400	0.963	49.000
AE	116.009	−133.738–365.756	0.363	49.000	2.006	0.308–3.705	**0.021**	49.000
Male	123.429	−129.498–376.356	0.339	49.000	0.453	−1.266–2.171	0.606	49.000
SC × Male	−280.910	−747.112–185.292	0.238	49.000	−1.686	−4.856–1.485	0.297	49.000
AE × Male	−322.897	−714.791–68.996	0.106	49.000	−1.657	−4.323–1.010	0.223	49.000
**Random Effects**								
σ^2^	100976.98				4.66			
τ_00_	89389.05 _Litter_				5.87 _Litter_			
ICC	0.47				0.56			
N	9 _Litter_				9 _Litter_			
Observations	57				57			
Marginal *R*^2^/Conditional *R*^2^	0.039/0.490				0.068/0.588			

**Table 5:** Regression table for Re→mPFC projection neurons, as visualized in Figure 2 and quantified in Figure 4. Table was generated using the “tab_model” function (df method = Satterthwaite) from the “sjPlot” package on “lmer” objects from the “lmerTest” package. Similar table controlling for injection properties (diffusion of AAVrg-CAG at the injection site (fixed effect), the amount of incubation time following AAV injection (fixed effect), and the fluorophore-site pairing (random effect)) can be found in the Supplementary Material (Section S.2.3). Significant predictors (i.e., *p* ≤ 0.050) are indicated by bold *p*-values.

	Number of Projection Neurons: Re→mPFC				% of toTal Re Neurons: Re→mPFC			
Predictors	Estimates	95% CI	*p*	*df*	Estimates	95% CI	*p*	*df*
(Intercept)	323.757	131.349–516.165	**0.001**	49.000	2.441	0.764–4.119	**0.004**	49.000
SC	10.852	−308.221–329.925	0.947	49.000	0.071	−2.779–2.922	0.961	49.000
AE	37.432	−245.785–320.649	0.796	49.000	1.036	−1.356–3.429	0.396	49.000
Male	−171.805	−461.872–118.261	0.246	49.000	−1.250	−3.697–1.197	0.317	49.000
SC × Male	110.667	−417.063–638.396	0.681	49.000	0.550	−3.910–5.010	0.809	49.000
AE × Male	129.919	−308.392–568.229	0.561	49.000	1.476	−2.238–5.190	0.436	49.000
**Random Effects**								
σ^2^	134920.91				9.57			
τ_00_	0.00 _Litter_				0.31 _Litter_			
ICC					0.03			
N	9 _Litter_				9 _Litter_			
Observations	57				57			
Marginal *R*^2^/Conditional *R*^2^	0.039/NA				0.073/0.102			

**Table 6:** Regression table for Re→vHPC projection neurons, as visualized in Figure 2 and quantified in Figure 4. Table was generated using the “tab_model” function (df method = Satterthwaite) from the “sjPlot” package on “lmer” objects from the “lmerTest” package. Similar table controlling for injection properties (diffusion of AAVrg-CAG at the injection site (fixed effect), the amount of incubation time following AAV injection (fixed effect), and the fluorophore-site pairing (random effect)) can be found in the Supplementary Material (Section S.2.3). Significant predictors (i.e., *p* ≤ 0.050) are indicated by bold *p*-values.

	Number of Projection Neurons: Re→vHPC				% of Total Re Neurons: Re→vHPC			
Predictors	Estimates	95% CI	*p*	*df*	Estimates	95% CI	*p*	*df*
(Intercept)	1885.348	584.630–3186.066	**0.004**	49.000	12.205	3.688–20.721	**0.005**	49.000
SC	1081.260	−1611.037–3773.558	0.431	49.000	8.259	−9.383–25.900	0.359	49.000
AE	−557.028	−1267.687–153.631	0.124	49.000	0.057	−4.523–4.636	0.981	49.000
Male	−160.655	−878.649–557.339	0.661	49.000	−0.626	−5.252–4.001	0.791	49.000
SC × Male	−503.139	−1829.473–823.195	0.457	49.000	−6.417	−14.964–2.130	0.141	49.000
AE × Male	281.193	−835.784–1398.170	0.622	49.000	3.208	−3.990–10.406	0.382	49.000
**Random Effects**								
σ^2^	812289.37				33.73			
τ_00_	2651619.58 _Litter_				114.25 _Litter_			
ICC	0.77				0.77			
N	9 _Litter_				9 _Litter_			
Observations	57				57			
Marginal *R*^2^/Conditional *R*^2^	0.074/0.783				0.055/0.785			

## Data Availability

The data presented in this study are available upon reasonable request from the corresponding author.

## Supplementary Materials

Supplementary materials for this experiment are available online at https://www.mdpi.com/2076-3425/11/3/323/s1. Figure S1: Weight of experimental animals throughout the current study. (a) Weight of animals throughout the early postnatal treatment period (postnatal days (PD) 4–9). Bold lines and symbols represent group means while lighter indi-vidual lines represent each individual animal. * *p* < 0.050 relative to SI procedural control group, # *p* < 0.050 relative to females of same treatment group, ## *p* < 0.010 relative to females of same treatment group, (b) Weight of animals at time of AAVrg-CAG injection in adulthood (PD 105-139). Black bars represent mean within treatment group with symbols representing each individual animal. ## *p* < 0.010 relative to females (main effect of sex), Table S1: Regression table for linear mixed-effects model examining weight during early postnatal treatment period (left) and in adulthood at time of AAVrg-CAG injection (right). Random effects for the treatment model were animal (random-intercept) and litter (random-intercept and ran-dom-slope across days of treatment). Litter was the only random effect in the model examining the adult timepoint (random-intercept), Figure S2: Quantification of all injection characteristics and incubation times. (a) Age at AA-Vrg-CAG injection and amount of incubation time post-injection (i.e., time between injection and tissue fixation); (b-d) Coordinates of mPFC injections along the anterior-posterior (AP) axis (b), the medial-lateral (ML) axis (c), and the dorsal-ventral (DV) axis (d). While the location of injec-tions between postnatal treatment groups was similar among the ML and DV axes, the AP coor-dinate of AE group appeared slightly further anterior relative to both control groups, likely due to reductions in frontal cortex volume following developmental AE. All coordinates are given in mm relative to Bregma; (e-g) Coordinates of vHPC injections along the AP (e), ML (f), and DV (g) ax-es. While the location of injections between postnatal treatment groups was similar among the AP and ML axes, the DV coordinate of AE group appeared slightly further ventral relative to both control groups, likely due to reductions in total brain volume following developmental AE. All coordinates are given in mm relative to Bregma; (h-i) Diffusion of AAVrg-CAG injections into mPFC (h) and vCA1 (i), quantified using unbiased stereological estimation (Cavalieri probe). For all panels in this figure, individual data points represent one animal and are superimposed over bars representing mean within that postnatal treatment. Data include both male and female ani-mals, which are not differentiated due to the lack of sex effects. Sample sizes are given in paren-theses below each treatment group. * *p* ≤ 0.050, ** *p* ≤ 0.010, Table S2: Regression table for linear mixed-effects model examining age at injection of AA-Vrg-CAG (left) and number of days between AAVrg-CAG injection and tissue fixation (right). Litter was the only random effect in both models (random-intercept), Table S3: Regression table for linear mixed-effects model examining stereotaxic coordinate of the center of AAVrg-CAG injection into mPFC along the anterior-posterior axis (left), medial-lateral axis (middle), and dorsal-ventral axis (right). Litter was the only random effect in at three models (random-intercept), Table S4: Regression table for linear mixed-effects model examining stereotaxic coordinate of the center of AAVrg-CAG injection into vHPC along the anterior-posterior axis (left), medial-lateral axis (middle), and dorsal-ventral axis (right). Litter was the only random effect in at three models (random-intercept), Table S5: Regression table for linear mixed-effects model examining diffusion of AAVrg-CAG in-jections into mPFC (left) and vHPC (right). Litter (random-intercept) and fluorophore-site pairing (random-intercept) were the random effects in both models, Table S6: Regression table for Re→(mPFC+vHPC) projection neurons, adding additional control predictors to the analysis from Table 4, Table S7: Regression table for Re→mPFC projection neurons, adding additional control predictors to the analysis from Table 5. Table S8: Regression table for Re→vHPC projection neurons, adding additional control predictors to the analysis from Table 5.
