# Supplementary material for: Changes in Representation of Thalamic Projection Neurons within Prefrontal-Thalamic-Hippocampal Circuitry in a Rat Model of Third Trimester Binge Drinking
Source: Brain Sci. 2021 Mar 4;11(3):323. doi: 10.3390/brainsci11030323 (PMC8001051; doi:10.3390/brainsci11030323)
Supplement: Supplementary file 1 [file brainsci-11-00323-s001.pdf]

brain sciences

MDPI

Supplementary Material

S.1. Analysis of weight during postnatal treatment period and in adulthood.

Weights of the AE treatment group diverged from control levels after the start of the treatment period (Figure S1a, Table S1). While such weight differences are commonly observed in studies of developmental alcohol exposure [32, 73, 74], weights no longer differed in adulthood (i.e., at time of AAVrg-CAG injection; Figure S1b, Table S1).

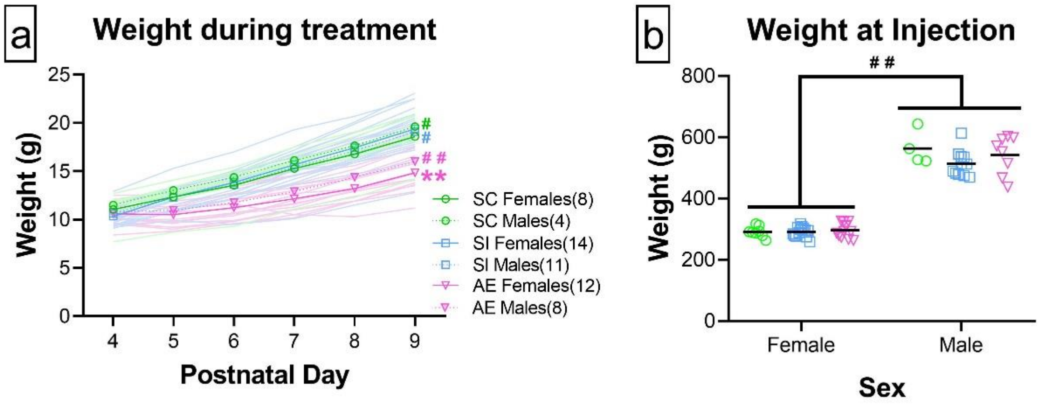

Figure S1. Weight of experimental animals throughout the current study. (**a**) Weight of animals throughout the early postnatal treatment period (postnatal days (PD) 4-9). Bold lines and symbols represent group means while lighter individual lines represent each individual animal. * p < 0.050 relative to SI procedural control group, # p < 0.050 relative to females of same treatment group, ## p < 0.010 relative to females of same treatment group, (**b**) Weight of animals at time of AAVrg-CAG injection in adulthood (PD 105-139). Black bars represent mean within treatment group with symbols representing each individual animal. ## p < 0.010 relative to females (main effect of sex).

Table S1. Regression table for linear mixed-effects model examining weight during early postnatal treatment period (left) and in adulthood at time of AAVrg-CAG injection (right). Random effects for the treatment model were animal (random-intercept) and litter (random-intercept and random-slope across days of treatment). Litter was the only random effect in the model examining the adult timepoint (random-intercept).

	Weight (PD4-9)				Weight (adulthood)			
Predictors	*Estimates*	*95% CI*	*p*	*df*	*Estimates*	*95% CI*	*p*	*df*
(Intercept)	10.52	9.64 to 11.40	<0.001	325.00	294.57	272.03 to 317.10	<0.001	49.00
SC	0.42	-1.27 to 2.11	0.624	325.00	3.54	-39.00 to 46.08	0.870	49.00
AE	-0.38	-1.30 to 0.54	0.421	325.00	7.31	-17.99 to 32.60	0.571	49.00
Male	0.10	-0.84 to 1.03	0.839	325.00	219.81	194.13 to 245.50	<0.001	49.00
# of days since PD4	**1.78**	**1.58** to **1.97**	**<0.001**	**325.00**				
SC * Male	0.32	-1.40 to 2.04	0.714	325.00	45.33	-1.88 to 92.54	0.060	49.00

AE * Male	0.05	-1.39 to 1.50	0.942	325.00	22.93	-16.67 to 62.52	0.256	49.00
SC * # of days since PD4	-0.29	-0.69 to 0.11	0.157	325.00				
AE * # of days since PD4	**-0.89**	**-1.00** to **-0.78**	**<0.001**	**325.00**				
Male * # of days since PD4	**-0.12**	**-0.24** to **-0.01**	**0.029**	**325.00**				
SC * Male * # of days since PD4	**0.24**	**0.04** to **0.45**	**0.021**	**325.00**				
AE * Male * # of days since PD4	**0.36**	**0.19** to **0.53**	**<0.001**	**325.00**				
Random Effects								
σ^2	0.34				1044.17			
τ_{00}	1.21 Animal<br>0.69 Litter				382.89 Litter			
τ_{11}	0.06 Litter.DaysSinceStartOfDosing							
ρ_{01}	0.12 Litter							
ICC	0.88				0.27			
N	9 Litter<br>57 Animal				9 Litter			
Observations	342				57			
Marginal R^2 / Conditional R^2	0.721 / 0.967				0.906 / 0.931			

S.2. Analysis of age of animals at injection, incubtion time of AAVrg-CAG prior to tissue fixation, injection locations, injection diffusion volumes.

S.2.1. Age at time of AAVrg-CAG injection and duration of AAVrg-CAG incubation prior to tissue fixation

Animals were counterbalanced such that there were no significant associations between either age at AAVrg-CAG injection or the amount of time between AAVrg-CAG injection and tissue perfusion and postnatal treatment or sex (Figure S2a, Table S2).

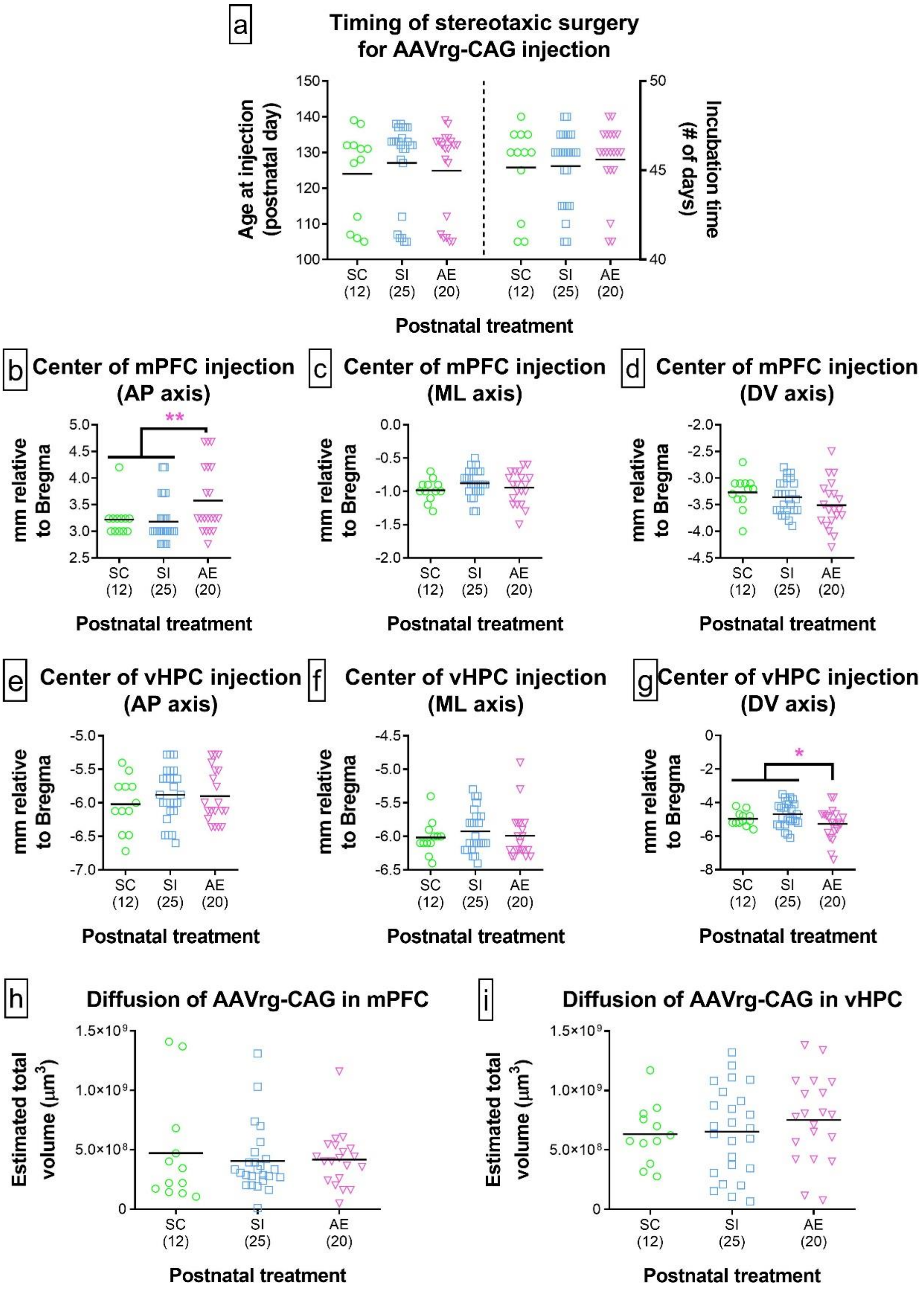

Figure S2. Quantification of all injection characteristics and incubation times. (**a**) Age at AAVrg-CAG injection and amount of incubation time post-injection (i.e., time between injection and tissue fixation); (**b-d**) Coordinates of mPFC injections along the anterior-posterior (AP) axis (**b**), the medial-lateral (ML) axis (**c**), and the dorsal-ventral (DV) axis (**d**). While the location of injections between postnatal treatment groups was similar among the ML and DV axes, the AP coordinate of AE group appeared slightly further anterior relative to both control groups, likely due to reductions in frontal cortex volume following developmental AE. All coordinates are given in mm relative to Bregma; (**e-g**) Coordinates of vHPC injections along the AP (**e**), ML (**f**), and DV (**g**) axes. While the location of injections between postnatal treatment groups was similar among the AP and ML axes, the DV coordinate of AE group appeared slightly further ventral relative to both

control groups, likely due to reductions in total brain volume following developmental AE. All coordinates are given in mm relative to Bregma; (**h-i**) Diffusion of AAVrg-CAG injections into mPFC (**h**) and vCA1 (**i**), quantified using unbiased stereological estimation (Cavalieri probe). For all panels in this figure, individual data points represent one animal and are superimposed over bars representing mean within that postnatal treatment. Data include both male and female animals, which are not differentiated due to the lack of sex effects. Sample sizes are given in parentheses below each treatment group. * p ≤0.050, ** p ≤0.010.

Table S2. Regression table for linear mixed-effects model examining age at injection of AAVrg-CAG (left) and number of days between AAVrg-CAG injection and tissue fixation (right). Litter was the only random effect in both models (random-intercept).

	**Age of AAVrg-CAG injection (postnatal day)**				**AAVrg-CAG incubation time (days)**			
Predictors	*Estimates*	*95% CI*	*p*	*df*	*Estimates*	*95% CI*	*p*	*df*
(Intercept)	128.1	121.0 to 135.2	**<0.001**	48.0	45.2	43.9 to 46.6	**<0.001**	48.0
SC	-4.4	-17.0 to 8.2	0.495	48.0	0.3	-2.1 to 2.8	0.790	48.0
AE	-0.9	-10.3 to 8.4	0.845	48.0	0.1	-1.3 to 1.6	0.870	48.0
Male	-1.5	-11.0 to 8.1	0.765	48.0	-0.2	-1.7 to 1.2	0.743	48.0
SC * Male	4.7	-12.8 to 22.2	0.596	48.0	-1.5	-4.2 to 1.2	0.266	48.0
AE * Male	-1.9	-16.5 to 12.7	0.797	48.0	-0.1	-2.3 to 2.2	0.950	48.0
Random Effects								
σ^2	145.53				3.35			
τ_{00}	17.14 Litter				1.35 Litter			
	0.00 mPFC_Fluorophore				0.11 mPFC_Fluorophore			
ICC					0.30			
N	2 mPFC_Fluorophore				2 mPFC_Fluorophore			
	9 Litter				9 Litter			
Observations	57				57			
Marginal R^2 / Conditional R^2	0.019 / NA				0.034 / 0.327			

S.2.2. Quantification of injection locations

The coordinates of every injection were identified based on the section which possessed the greatest expression of AAV-induced fluorescence. First, the location along the anterior-posterior axis was identified by matching the section to the most similar plate in Paxinos and Watson [75]. The coordinates along the medial-lateral and dorsal-ventral axes were calculated using the relative distance between the midline and most lateral point of the section, and the most dorsal and ventral points of the section, and confirmed by visual inspection using relative location compared to common landmarks (e.g., forceps minor of the corpus collosum and olfactory bulb in PFC; suprapyramidal blade of the dentate gyrus and rhinal sulcus in HPC).

Representative injections of AAVrg-CAG are presented in Figure 2, accompanied by examples of retrograde labeling in Re. The pairing between injection site and fluorophore

(i.e., injection of AAVrg-CAG-tdTomato versus AAVrg-CAG-GFP into mPFC or vHPC) were counterbalanced between treatment groups.

Injections into the mPFC had similar medial-lateral (ML) and dorsal-ventral (DV) coordinates across all treatment groups. The mPFC injections in the AE treatment group appeared further anterior in the brain relative to control groups, likely due to reductions in total brain volume frequently observed following developmental AE in both humans [5, 76] and rats [30,31]. Regardless of this average difference in anterior-posterior (AP) coordinate relative to major brain landmarks, injections were still centered along the dorsal boundary of prelimbic mPFC in all treatment groups. These data are presented in Figure S2b-S2d and Table S3.

While there is evidence of inhomogeneity of thalamic innervation of PFC [77], AP coordinate of mPF injection was not significantly correlated with either the number or percentage of Re→(mPFC+vHPC) neurons (p's = 0.864 and 0.693, respectively) or the number or percentage of Re→mPFC neurons (p's = 0.616 and 0.636, respectively).

Table S3. Regression table for linear mixed-effects model examining stereotaxic coordinate of the center of AAVrg-CAG injection into mPFC along the anterior-posterior axis (left), medial-lateral axis (middle), and dorsal-ventral axis (right). Litter was the only random effect in at three models (random-intercept).

	**Center of mPFC injection: anterior-posterior coordinate (mm relative to Bregma)**				**Center of mPFC injection: medial-lateral coordinate (mm relative to Bregma)**				**Center of mPFC injection: dorsal-ventral coordinate (mm relative to Bregma)**			
Predictors	*Estimates*	*95% CI*	*p*	*df*	*Estimates*	*95% CI*	*p*	*df*	*Estimates*	*95% CI*	*p*	*df*
(Intercept)	3.25	2.98 to 3.52	**<0.001**	49.00	-0.90	-1.02 to -0.79	**<0.001**	49.00	-3.42	-3.64 to -3.20	**<0.001**	49.00
SC	0.02	-0.47 to 0.51	0.945	49.00	-0.09	-0.30 to 0.11	0.372	49.00	0.19	-0.22 to 0.61	0.356	49.00
AE	0.53	0.18 to 0.87	**0.003**	49.00	-0.05	-0.21 to 0.11	0.560	49.00	-0.12	-0.37 to 0.13	0.352	49.00
Male	-0.13	-0.49 to 0.22	0.457	49.00	0.05	-0.11 to 0.22	0.542	49.00	0.18	-0.08 to 0.44	0.168	49.00
SC * Male	-0.01	-0.66 to 0.64	0.969	49.00	-0.03	-0.34 to 0.27	0.846	49.00	-0.18	-0.65 to 0.30	0.467	49.00
AE * Male	-0.37	-0.91 to 0.17	0.180	49.00	-0.04	-0.30 to 0.21	0.743	49.00	0.02	-0.38 to 0.41	0.937	49.00
Random Effects												
σ^2	0.20				0.04				0.10			
τ_{00}	0.03 $_{Litter}$				0.00 $_{Litter}$				0.03 $_{Litter}$			
ICC	0.14				0.06				0.24			
N	9 $_{Litter}$				9 $_{Litter}$				9 $_{Litter}$			
Observations	57				57				57			
Marginal R^2 / Conditional R^2	0.199 / 0.313				0.046 / 0.105				0.096 / 0.317			

Injections into the vHPC had similar AP and ML coordinates across all treatment groups (p's>0.190). The vHPC injections in the AE treatment group appeared further

ventral in the brain relative to control groups (p=0.014), likely due to reductions in total brain volume frequently observed following developmental AE in both humans [5, 76] and rats [30, 31]. Regardless of this average difference in DV coordinate relative to major brain landmarks, injections were still centered ventral CA1 in all treatment groups. These data are presented in Figure S2e-S2g and Table S4

Table S4. Regression table for linear mixed-effects model examining stereotaxic coordinate of the center of AAVrg-CAG injection into vHPC along the anterior-posterior axis (left), medial-lateral axis (middle), and dorsal-ventral axis (right). Litter was the only random effect in at three models (random-intercept).

	**Center of vHPC injection: anterior-posterior coordinate (mm relative to Bregma)**				**Center of vHPC injection: medial-lateral coordinate (mm relative to Bregma)**				**Center of vHPC injection: dorsal-ventral coordinate (mm relative to Bregma)**			
Predictors	*Estimates*	*95% CI*	*p*	*df*	*Estimates*	*95% CI*	*p*	*df*	*Estimates*	*95% CI*	*p*	*df*
(Intercept)	-5.94	-6.14 to -5.74	**<0.001**	49.00	-6.02	-6.20 to -5.84	**<0.001**	49.00	-4.90	-5.37 to -4.44	**<0.001**	49.00
SC	-0.21	-0.55 to 0.13	0.225	49.00	0.04	-0.28 to 0.36	0.809	49.00	-0.04	-0.91 to 0.83	0.935	49.00
AE	0.10	-0.20 to 0.40	0.515	49.00	-0.09	-0.32 to 0.14	0.456	49.00	-0.68	-1.21 to -0.14	**0.014**	49.00
Male	0.14	-0.17 to 0.45	0.387	49.00	0.19	-0.04 to 0.43	0.110	49.00	0.40	-0.15 to 0.95	0.150	49.00
SC * Male	0.25	-0.31 to 0.82	0.376	49.00	-0.29	-0.72 to 0.14	0.190	49.00	-0.51	-1.51 to 0.49	0.318	49.00
AE * Male	-0.28	-0.75 to 0.19	0.237	49.00	0.06	-0.30 to 0.43	0.726	49.00	0.28	-0.57 to 1.12	0.521	49.00
**Random Effects**												
σ^2	0.15				0.09				0.47			
τ_{00}	0.00 Litter				0.01 Litter				0.15 Litter			
ICC					0.13				0.24			
N	9 Litter				9 Litter				9 Litter			
Observations	57				57				57			
Marginal R^2 / Conditional R^2	0.085 / NA				0.102 / 0.218				0.167 / 0.366			

There were no significant differences in the diffusion of AAVrg-CAG in mPFC (Figure S2h) or vHPC (Figure S2i) between treatment groups or sexes. These results are presented in Table S5.

Table S5. Regression table for linear mixed-effects model examining diffusion of AAVrg-CAG injections into mPFC (left) and vHPC (right). Litter (random-intercept) and fluorophore-site pairing (random-intercept) were the random effects in both models.

	**Diffusion of AAVrg-CAG in mPFC (μm³)**	**Diffusion of AAVrg-CAG in vHPC (μm³)**

Predictors	*Estimates*	*95% CI*	*p*	*df*	*Estimates*	*95% CI*	*p*	*df*
(Intercept)	407473018	212969499 to 601976537	**<0.001**	48	590400000	407207584 to 773592416	**<0.001**	48
SC	99029812	-266135073 to 464194697	0.595	48	15600000	-288190254 to 319390254	0.920	48
AE	1160682	-221202390 to 223523754	0.992	48	190400000	-79251935 to 460051935	0.166	48
Male	39487739	-186416705 to 265392183	0.732	48	145309091	-130863868 to 421482049	0.302	48
SC * Male	58809432	-356237209 to 473856072	0.781	48	-62509091	-564961750 to 439943568	0.807	48
AE * Male	71696599	-276304082 to 419697279	0.686	48	-212109091	-629425835 to 205207653	0.319	48
**Random Effects**								
σ^2		80786528076139248.00				122305738609623232.00		
τ_{00}		27004016396804432.00 $_{Litter}$				3236.26 $_{Litter}$		
		0.00 $_{mPFC\_Fluorophore}$				0.00 $_{vHPC\_Fluorophore}$		
N		2 $_{mPFC\_Fluorophore}$				2 $_{vHPC\_Fluorophore}$		
		9 $_{Litter}$				9 $_{Litter}$		
Observations		57				57		
Marginal R^2 / Conditional R^2		0.043 / NA				0.045 / NA		

S.2.3. Analysis of AE-induced changes in Re→(mPFC+vHPC) projection neuron representation after controlling for injection properties

The current study found that AE increased the proportion of Re→(mPFC+vHPC) projection neurons, but not Re neurons projecting to either mPFC or vHPC alone. We confirmed this finding by running the same analyses in Tables 4-6 and controlling for the incubation time (fixed-effect), diffusion of AAVrg-CAG in each respective injection site (fixed effects), and fluorophore-injection site pairing (random-intercept). These analyses show similar statistical outcomes regardless of whether injection properties were controlled for, and are presented in Tables S6-S8, below.

Table S6. Regression table for Re→ (mPFC+vHPC) projection neurons, adding additional control predictors to the analysis from Table 4.

	**# of projection neurons: Re→(mPFC+vHPC) (controlling for injection parameters)**				**% of total Re neurons: Re→(mPFC+vHPC) (controlling for injection parameters)**			
Predictors	*Estimates*	*95% CI*	*p*	*df*	*Estimates*	*95% CI*	*p*	*df*
(Intercept)	585.514	-1986.959 to 3157.986	0.656	44.000	-6.697	-23.955 to 10.560	0.447	44.000
SC	43.604	-537.358 to 624.566	0.883	44.000	0.464	-3.900 to 4.827	0.835	44.000

AE	119.294	-141.061 to 379.649	0.369	44.000	2.271	0.566 to 3.976	**0.009**	44.000
Male	135.680	-128.562 to 399.921	0.314	44.000	0.938	-0.791 to 2.668	0.287	44.000
IncubationTime	-4.175	-60.613 to 52.264	0.885	44.000	0.209	-0.169 to 0.587	0.279	44.000
mPFC_Diffusion	-64.300	-177.378 to 48.778	0.265	44.000	-0.043	-0.800 to 0.713	0.911	44.000
vHPC_Diffusion	-1.840	-98.457 to 94.778	0.970	44.000	-0.517	-1.150 to 0.117	0.110	44.000
SC * Male	-281.139	-763.418 to 201.139	0.253	44.000	-1.551	-4.709 to 1.607	0.336	44.000
AE * Male	-325.318	-744.785 to 94.150	0.128	44.000	-2.541	-5.290 to 0.208	0.070	44.000
mPFC_Diffusion * vHPC_Diffusion	-13.328	-111.713 to 85.057	0.791	44.000	-0.507	-1.154 to 0.140	0.124	44.000
Random Effects								
σ^2	104323.44				4.46			
τ_{00}	99091.74 $_{Litter}$				6.09 $_{Litter}$			
	0.00 $_{mPFC\_Fluorophore}$				0.00 $_{mPFC\_Fluorophore}$			
ICC					0.58			
N	2 $_{mPFC\_Fluorophore}$				2 $_{mPFC\_Fluorophore}$			
	9 $_{Litter}$				9 $_{Litter}$			
Observations	57				57			
Marginal R^2 / Conditional R^2	0.108 / NA				0.116 / 0.626			

Table S7. Regression table for Re→mPFC projection neurons, adding additional control predictors to the analysis from Table 5.

	**# of projection neurons: Re→mPFC (controlling for injection parameters)**				**% of total Re neurons: Re→mPFC (controlling for injection parameters)**			
Predictors	*Estimates*	*95% CI*	*p*	*df*	*Estimates*	*95% CI*	*p*	*df*
(Intercept)	-697.215	-2723.583 to 1329.152	0.500	46.000	-7.713	-24.556 to 9.130	0.369	46.000
SC	23.048	-248.817 to 294.914	0.868	46.000	0.179	-2.074 to 2.431	0.876	46.000
AE	-17.708	-259.398 to 223.982	0.886	46.000	0.520	-1.482 to 2.523	0.610	46.000
Male	-129.263	-376.611 to 118.086	0.306	46.000	-0.933	-2.983 to 1.116	0.372	46.000
IncubationTime	22.653	-21.187 to 66.493	0.311	46.000	0.227	-0.137 to 0.590	0.222	46.000
mPFC_Diffusion	-85.183	-172.772 to 2.407	0.057	46.000	-0.719	-1.445 to 0.007	0.052	46.000
SC * Male	205.484	-250.987 to 661.955	0.378	46.000	1.430	-2.351 to 5.212	0.459	46.000

AE * Male	190.927	-182.271 to 564.125	0.316	46.000	2.126	-0.965 to 5.218	0.178	46.000
Random Effects								
σ^2	97188.46				6.67			
τ_{00}	0.00 Litter				0.00 Litter			
	42893.70 mPFC_Fluorophore				3.55 mPFC_Fluorophore			
N	2 mPFC_Fluorophore				2 mPFC_Fluorophore			
	9 Litter				9 Litter			
Observations	57				57			
Marginal R^2 / Conditional R^2	0.111 / NA				0.159 / NA			

Table S8. Regression table for Re→vHPC projection neurons, adding additional control predictors to the analysis from Table 5.

	**# of projection neurons: Re→vHPC (controlling for injection parameters)**				**% of total Re neurons: Re→vHPC (controlling for injection parameters)**			
Predictors	*Estimates*	*95% CI*	*p*	*df*	*Estimates*	*95% CI*	*p*	*df*
(Intercept)	8012.884	1521.502 to 14504.266	**0.016**	46.000	2.241	-34.641 to 39.123	0.905	46.000
SC	1024.337	-1066.736 to 3115.410	0.337	46.000	7.715	-6.303 to 21.734	0.281	46.000
AE	-494.043	-1179.397 to 191.311	0.158	46.000	1.710	-2.069 to 5.490	0.375	46.000
Male	-203.009	-887.162 to 481.143	0.561	46.000	-0.011	-3.780 to 3.758	0.996	46.000
IncubationTime	-138.187	-278.445 to 2.070	0.053	46.000	0.183	-0.595 to 0.961	0.644	46.000
vHPC_Diffusion	44.844	-207.682 to 297.370	0.728	46.000	-1.207	-2.601 to 0.188	0.090	46.000
SC * Male	-673.099	-1928.386 to 582.188	0.293	46.000	-6.620	-13.538 to 0.297	0.061	46.000
AE * Male	142.666	-926.204 to 1211.537	0.794	46.000	0.603	-5.295 to 6.501	0.841	46.000
Random Effects								
σ^2	710794.91				21.55			
τ_{00}	1521485.90 Litter				71.99 Litter			
	314428.91 vHPC_Fluorophore				35.96 vHPC_Fluorophore			
ICC	0.72				0.83			
N	2 vHPC_Fluorophore				2 vHPC_Fluorophore			
	9 Litter				9 Litter			
Observations	57				57			

Marginal R^2 / Conditional R^2	0.111 / 0.752	0.061 / 0.844
